# Treatment of Acute Grade I Proximal Femoral Epiphysiolysis With Hip-Preserving Surgery: An Arthroscopic Approach

**DOI:** 10.7759/cureus.116915

**Published:** 2026-09-25

**Authors:** Lucas Cortizo Garcia, Davi Araújo Veiga Rosário, Fernando Cal Garcia Filho, Mariana Cantao, Thales Felipe Lucas Sena, Daniel Mascarenhas Isaac

**Affiliations:** 1 Orthopedics, Clinica Ortoped, Salvador, BRA

**Keywords:** arthroscopy, femoracetabular impingement, femur head, hip joint, slipped capital femoral epiphyses

## Abstract

Slipped capital femoral epiphysis (SCFE) is the most common hip disorder in adolescence. Although in situ fixation remains the standard treatment for stable slips, it does not correct metaphyseal prominence. Open reconstructive procedures may better restore anatomy but with greater morbidity. This article describes an arthroscopic hip-preserving surgical technique for the treatment of acute Grade I SCFE. Patients were positioned supine on a traction table with padded boots and a well-padded perineal post. Under fluoroscopic guidance, in situ fixation was performed using a partially threaded 7.0-mm cannulated screw placed perpendicular to the physis. Standard anterolateral and mid-anterior portals were established, followed by synovectomy, interportal capsulotomy, and arthroscopic evaluation. The retinacular vessels were identified and preserved. A T-shaped capsulotomy allowed exposure of the head-neck junction. Cam deformity was defined after periosteal resection, and femoral osteoplasty was performed with a high-speed burr under fluoroscopic and arthroscopic control. Dynamic assessment confirmed adequate resection and the absence of residual impingement. Arthroscopic treatment with in situ fixation represents a potential hip-preserving approach for acute Grade I SCFE. Further studies are needed to determine long-term safety, functional outcomes, and the potential to prevent early degenerative changes.

## Introduction

Slipped capital femoral epiphysis (SCFE) is the most common hip disorder of adolescence and results from displacement through the proximal femoral physis, with the epiphysis remaining relatively seated in the acetabulum while the metaphysis displaces [1]. Its incidence varies widely across populations, reflecting differences in age, sex, ethnicity, and obesity-related risk [2]. Beyond the acute slip, the major concern is the residual proximal femoral deformity, which alters head-neck offset, promotes cam-type femoroacetabular impingement, and may initiate early cartilage and labral damage [3]. For that reason, SCFE should be viewed not only as a physeal injury but also as a precursor of mechanical hip dysfunction and premature osteoarthritis. In this report, Grade I SCFE refers to a mild slip according to the Southwick classification. The slip was also classified as stable according to the Loder criteria.

In situ fixation remains the standard treatment for most stable slips because it reliably halts further displacement with relatively low immediate morbidity [3]. However, stabilization alone does not correct the metaphyseal prominence caused by the slip, and many patients continue to exhibit impingement morphology after healing [4]. This residual deformity has been associated with pain, restricted range of motion, and early chondrolabral injury, even in apparently mild slips [4,5]. Although open reconstructive procedures can restore proximal femoral anatomy more aggressively, they also introduce greater surgical morbidity, higher technical demands, and a more substantial complication profile, which has intensified interest in less invasive hip-preserving strategies.

As hip arthroscopy has evolved from a diagnostic adjunct to a primary therapeutic tool in adolescents, its role in SCFE-related deformity has expanded accordingly [6]. Arthroscopy allows simultaneous assessment of intra-articular damage and evaluation of cam morphology and provides a less invasive approach, which is especially attractive when vascular preservation is a central concern in hip-preservation surgery [7]. Early reports suggest that arthroscopic osteoplasty may improve impingement-related morphology in selected patients with mild-to-moderate SCFE. However, evidence supporting simultaneous correction of the metaphyseal prominence during the acute treatment of low-grade SCFE remains limited [8,9]. Therefore, the purpose of this article is to describe a reproducible hip-preserving arthroscopic technique for the treatment of acute Grade I SCFE.

## Technical report

Surgical technique

For the arthroscopic procedure, patients were positioned supine on an orthopedic traction table. The feet were placed in padded boots, and a large, well-padded perineal post was used to ensure stability and minimize the risk of soft tissue injury. An arthroscopy pump system was routinely employed to maintain adequate joint distension and visualization.

Under fluoroscopic guidance, in situ fixation was performed using a partially threaded 7.0-mm cannulated screw (4-thread lead, 16-mm thread length). The screw was inserted perpendicular to the physis, ensuring accurate positioning and preservation of adjacent anatomical structures, including the epiphyseal tubercle. Fluoroscopy provided real-time imaging to confirm correct placement and reduce the risk of iatrogenic injury.

Following fixation, standard anterolateral (AL) and mid-anterior (MA) portals were established, as previously described in the literature. A complete synovectomy was performed using a motorized shaver, followed by an interportal capsulotomy extending from the MA to the AL portal. The capsular edges were debrided with a soft tissue shaver to optimize visualization (Figure 1).

**Figure 1:**
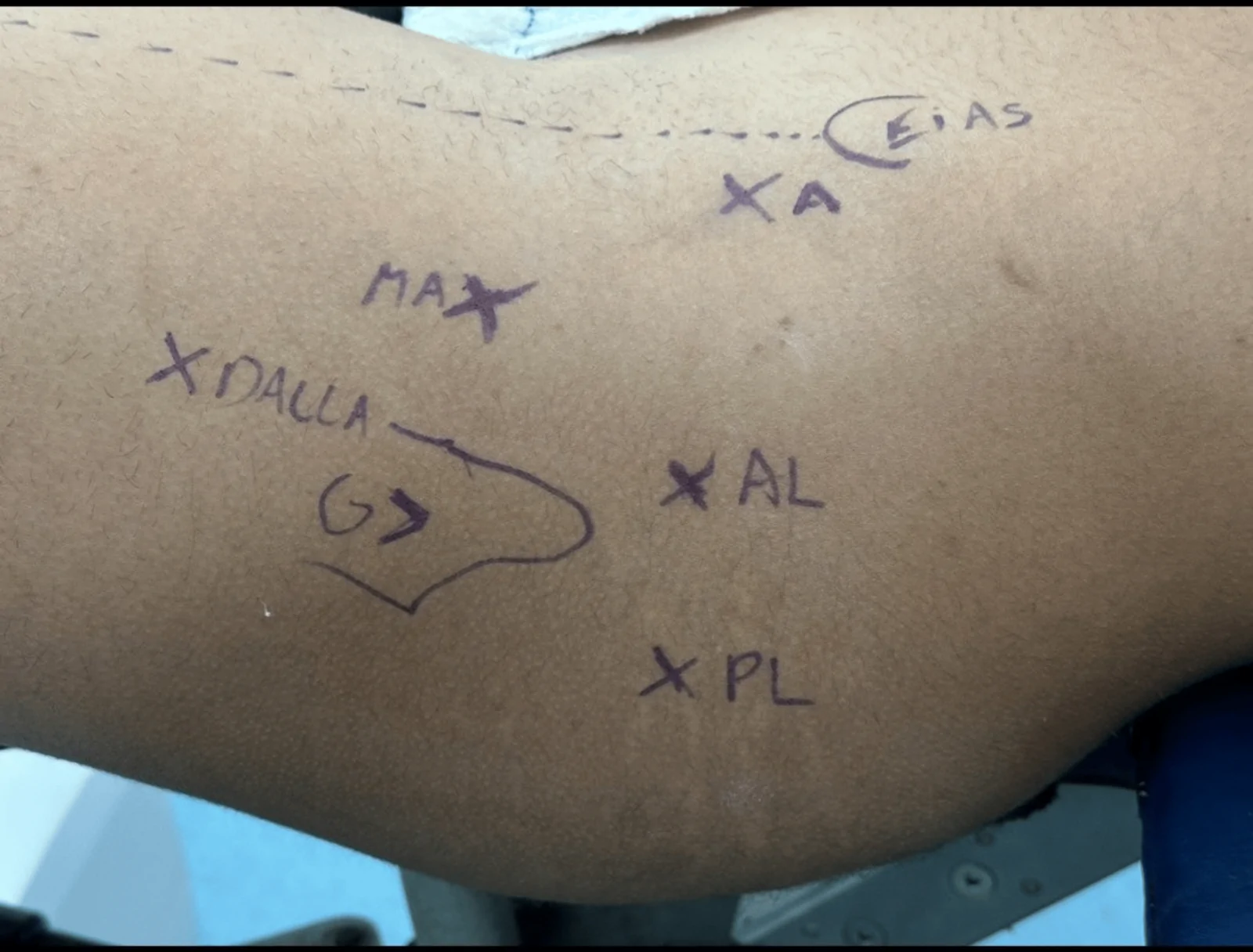
Surface anatomical landmarks and arthroscopic portal placement for hip arthroscopy. Anterior superior iliac spine (ASIS), anterior (A), anterolateral (AL), posterolateral (PL), distal anterolateral accessory (DALA), greater trochanter (GT), and mid-anterior (MA) portals.

A systematic arthroscopic evaluation of the central compartment was then conducted (Figure 2). No chondrolabral lesions or ligamentum teres injuries were identified. With the arthroscope positioned in the MA portal, attention was directed to the peripheral compartment (Figure 3). The retinacular branches of the medial femoral circumflex artery were carefully identified and preserved, with traction and controlled hip extension maintained to optimize visualization.

**Figure 2:**
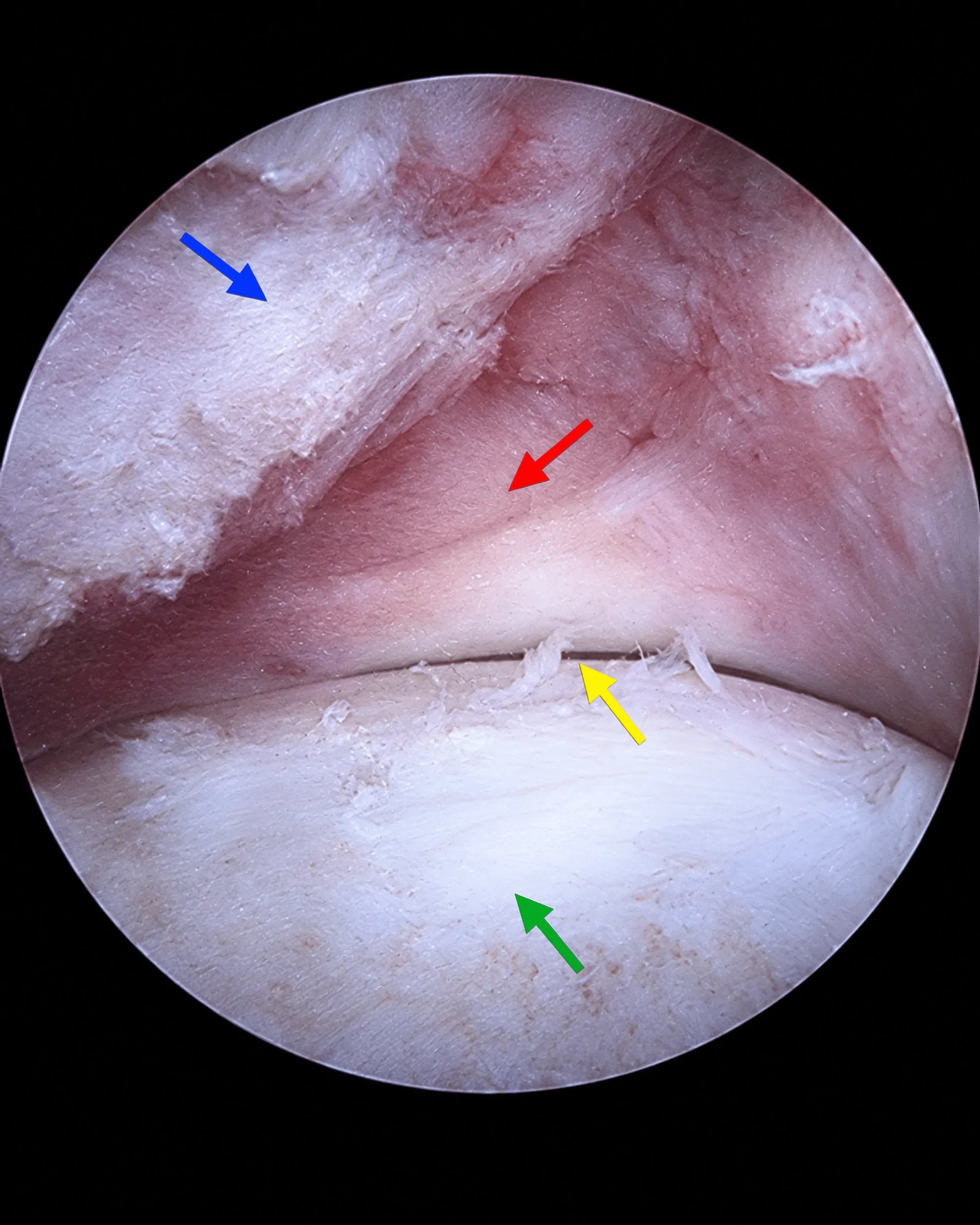
Arthroscopic view of the central compartment demonstrating the acetabular labrum (red arrow), articular capsule (blue arrow), proximal femoral physis (yellow arrow), and femoral epiphysis (green arrow).

**Figure 3:**
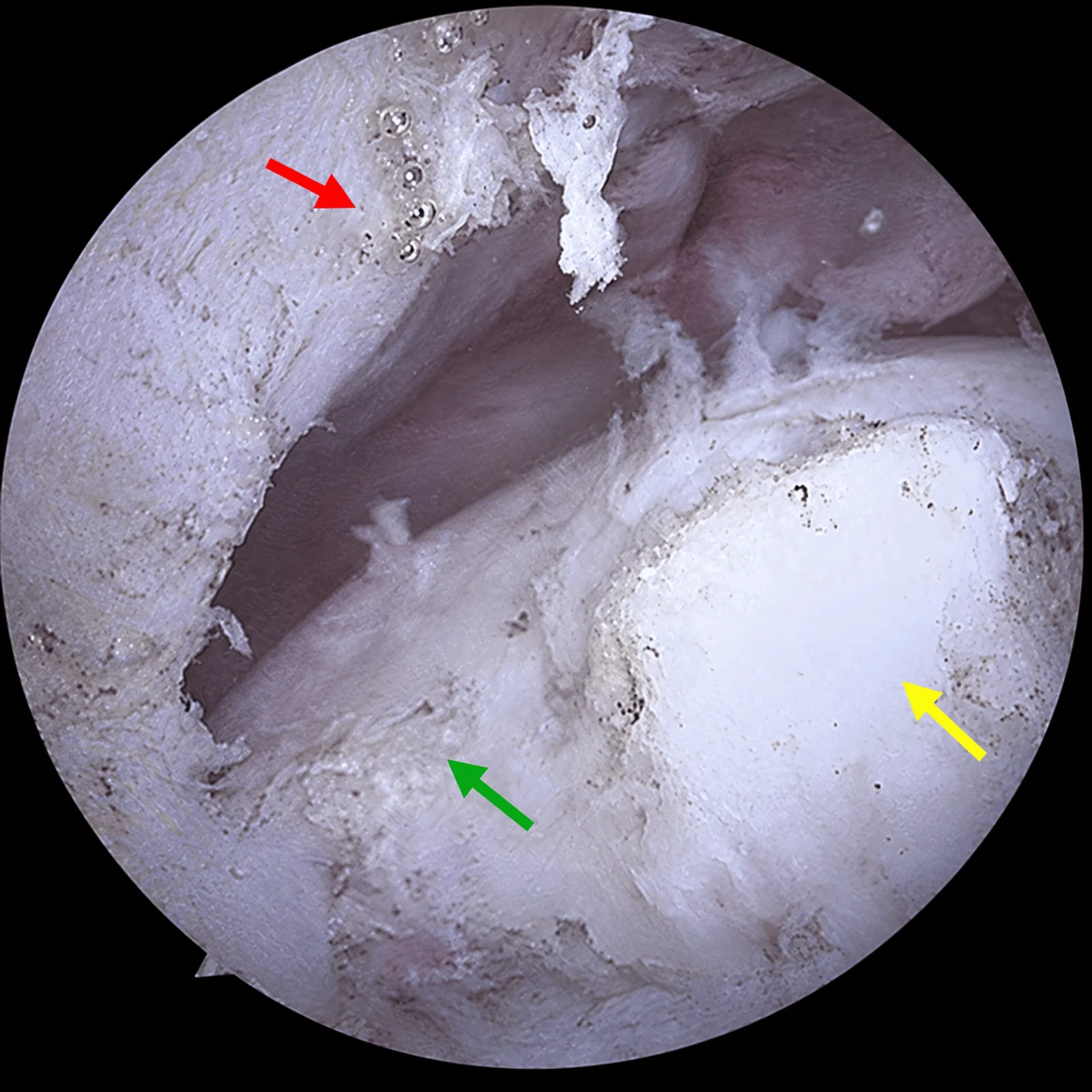
Arthroscopic view of the peripheral compartment demonstrating the superior articular capsule (red arrow), femoral epiphysis (green arrow), and metaphyseal prominence associated with the slip deformity (yellow arrow).

The capsulotomy was then extended along the anterolateral femoral neck in a T-shaped configuration. This step was performed cautiously to avoid injury to the retinacular vessels. The shaver was advanced toward the intertrochanteric line, allowing improved exposure of the head-neck junction for deformity assessment.

The anterior femoral neck periosteum was carefully resected using a bone shaver to better define the cam deformity (Figures 4, 5). Femoral osteoplasty was then performed to address femoroacetabular impingement. A high-speed burr with a 5.5 mm hood was introduced through the AL portal to perform anterosuperior head-neck reshaping.

**Figure 4:**
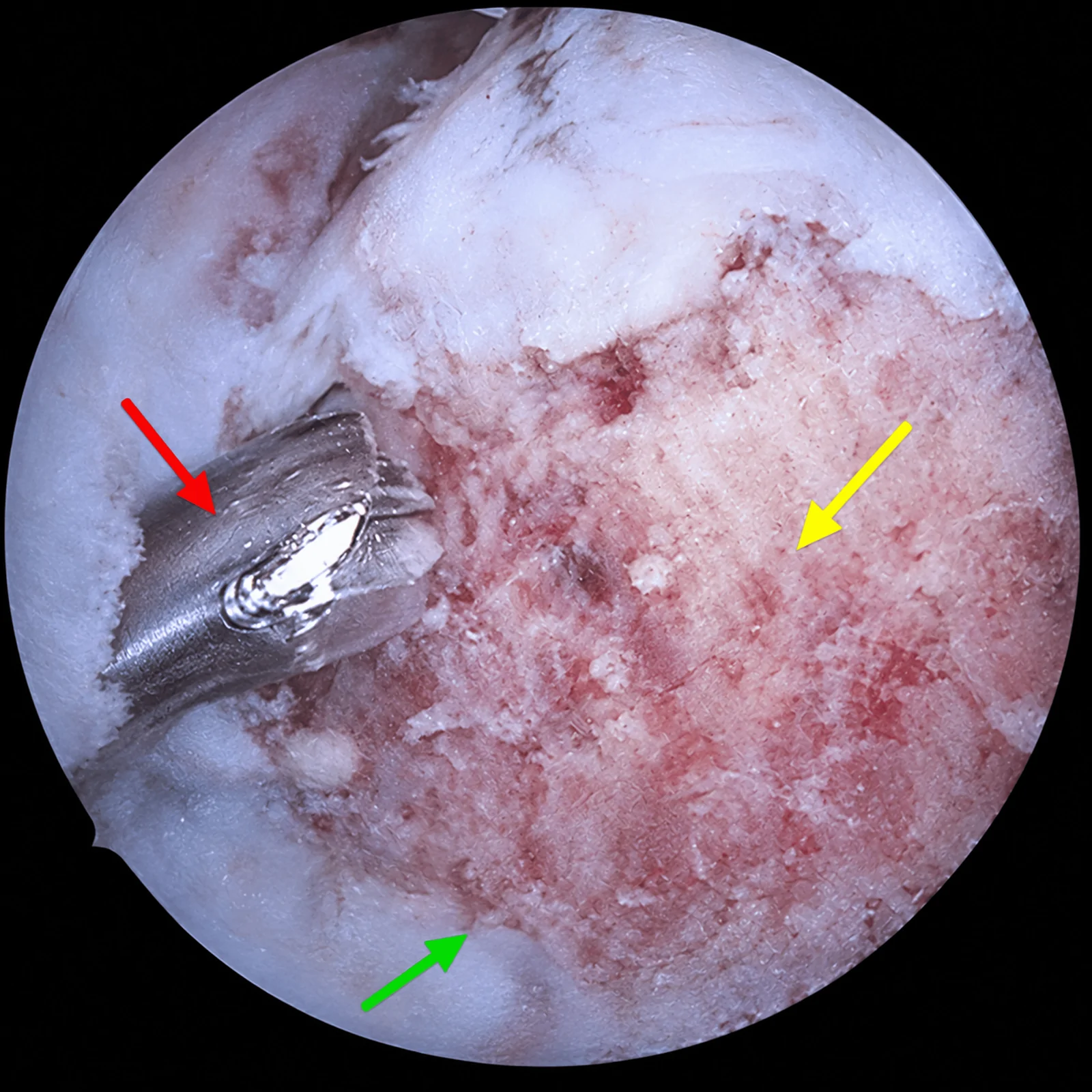
Arthroscopic view during osteoplasty showing the shaver (red arrow), the resected metaphyseal prominence adjacent to the proximal femoral physis (yellow arrow), and the femoral epiphysis (green arrow).

**Figure 5:**
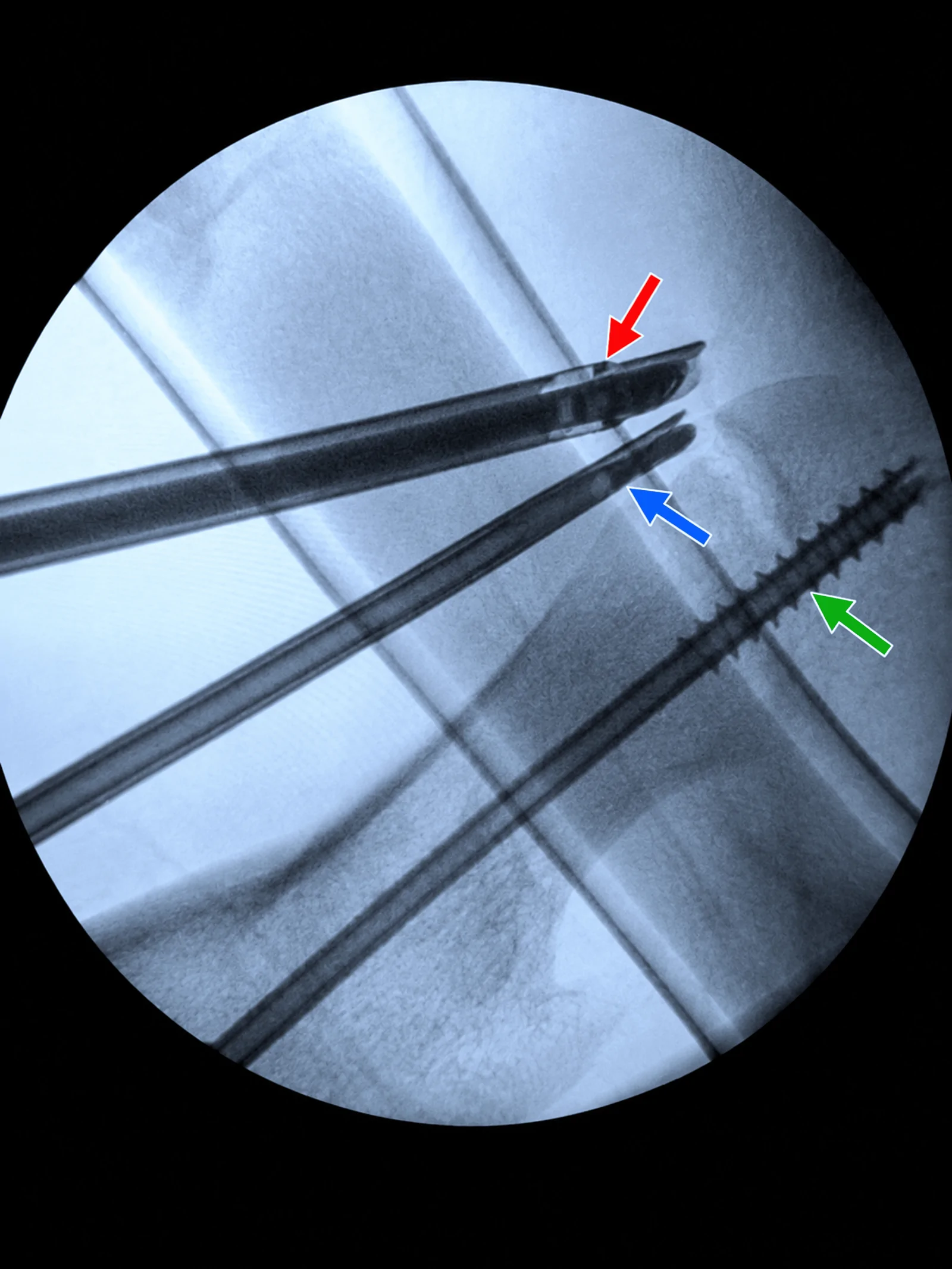
Fluoroscopic view demonstrating the arthroscope (red arrow), arthroscopic instrument used for resection of the proximal femoral metaphyseal prominence (blue arrow), and in situ fixation with a 7.0-mm cannulated screw (green arrow).

Unlike conventional arthroscopy for idiopathic femoroacetabular impingement, this technique often requires hip hyperextension to adequately expose the proximal metaphysis. Fluoroscopy was used to identify the physis and define a safe starting point for osteoplasty. Intermittent fluoroscopic imaging ensured adequate bone resection, while direct visualization allowed continuous monitoring of retinacular vessel integrity.

With the arthroscope in the MA portal, the anteroinferior margin of the deformity was identified. The inferior retinaculum of Weitbrecht was used as a landmark to define the distal limit of resection, which was performed up to the level of the physis. Adequacy of osteoplasty and restoration of femoral head-neck offset were confirmed fluoroscopically.

Dynamic assessment with flexion and extension under direct visualization was performed to confirm the absence of residual impingement. At the end of the procedure, the arthroscope was removed, and the portals were closed with absorbable sutures.

Postoperative protocol

Postoperatively, patients were instructed to restrict weight-bearing using crutches with toe-touch support for 12 weeks. From postoperative day 4, rehabilitation included daily 20-minute stationary cycling, muscle activation exercises, and gradual range-of-motion progression during the first 30 days.

Between 30 and 90 days, emphasis was placed on improving range of motion and muscle strength. After 90 days, gait training and proprioceptive exercises were initiated, progressing to full weight-bearing. Rehabilitation intensity was carefully monitored to avoid hip flexor tendinitis.

Return to unrestricted physical activity was allowed after six months. Follow-up included suture removal at 21 days and monthly radiographic evaluation. At one year, a three-dimensional computed tomography scan was obtained to assess remodeling and deformity correction.

## Discussion

The rationale for this technique is based on the fact that in situ fixation stabilizes the physis but does not fully neutralize the mechanical consequences of the slip. This limitation is consistent with the literature showing that fixation alone frequently leaves a residual metaphyseal prominence, with persistent loss of head-neck offset and a structural predisposition to cam-type impingement. Millis and Novais (2011) [10] emphasized that femoroacetabular impingement must be considered in every SCFE hip, even in mild slips, while Hosalkar et al. (2012) [4] linked this morphology to acetabular-neck conflict and cartilage injury. Long-term series after in situ screw fixation have likewise shown radiographic signs of impingement years after apparently successful stabilization, suggesting that physeal control and mechanical restoration are not equivalent goals [11].

The described technique is based on the concept that even low-grade SCFE may justify correction of the residual cam deformity at the index procedure. This view aligns with reports showing that minor slips can still produce substantial chondrolabral damage, pain, loss of internal rotation, and early degenerative change, particularly when head-neck offset remains insufficient [7]. In contrast to isolated pinning, authors advocating combined correction argue that simultaneous osteoplasty may reduce persistent impingement and avoid a second reconstructive stage. Chen et al. (2014) [8] reported improved radiographic and motion parameters after arthroscopic osteoplasty, and Leunig et al. (2017) [12] argued that pinning in situ in minor slips should be paired with recreation of anterior metaphyseal waisting, underscoring that “mild” does not necessarily mean mechanically benign.

From a technical perspective, arthroscopy may provide a less invasive approach for selected low-grade SCFE. Compared with surgical hip dislocation and proximal femoral realignment procedures, arthroscopy avoids trochanteric osteotomy and extensive soft-tissue dissection while still permitting assessment of both the central and peripheral compartments and simultaneous treatment of cam deformity and intra-articular pathology [13]. Recent reviews have shown functional and radiographic improvement after arthroscopic osteoplasty in SCFE-related impingement, whereas open procedures remain more anatomically corrective but inherently more morbid and technically demanding [8,14]. In this context, arthroscopy appears particularly attractive when the deformity is limited and the main objective is impingement control rather than full capital realignment.

Another relevant point is that vascular preservation is the central technical issue in any correction around the proximal femoral neck in SCFE. The literature on open corrective strategies, especially surgical dislocation and the modified Dunn procedure, makes clear that restoration of anatomy comes at the cost of strict dependence on meticulous handling of the retinacular flap because osteonecrosis remains the complication of greatest concern when vascular tension or injury occurs [15,16]. Ganz-based approaches were specifically developed around preservation of the femoral head blood supply, and contemporary modified Dunn reports still stress expert knowledge of retinacular anatomy as a prerequisite for safe correction [16,17]. Against this background, direct arthroscopic identification and protection of the retinacular vessels in our technique is not a minor detail but a fundamental rationale for choosing a less aggressive hip-preserving strategy in acute low-grade slips.

This technique appears best suited to acute, low-grade SCFE rather than the full spectrum of slip severity. This position is consistent with the literature, which generally reserves arthroscopic osteoplasty for mild-to-moderate, stable deformities, whereas more severe or unstable slips are more often managed with open realignment procedures because arthroscopy may be insufficient to restore anatomy in advanced displacement [6]. Reviews of pediatric hip arthroscopy and technical reports on post-SCFE deformity repeatedly frame arthroscopy as a selective strategy, not a universal solution [18,19]. Accordingly, extrapolating our approach to unstable or high-grade SCFE would not be supported by the current evidence base.

Arthroscopic inspection also highlights the diagnostic value of direct joint assessment, even when no chondrolabral damage is identified. Prior arthroscopic series in mild-to-moderate SCFE have shown that acetabular cartilage lesions, labral hyperemia or fraying, and other intra-articular abnormalities are common, indicating that structural injury may already be present even in relatively limited deformities [20]. Wylie et al. (2015) [21] reported cartilage and/or labral damage in symptomatic post-SCFE hips, and Accadbled et al. (2017) [18] similarly emphasized that arthroscopy often reveals acetabular and labral abnormalities. In contrast, the absence of chondrolabral lesions in our case may reflect the acute presentation and low-grade morphology, which potentially limited cumulative mechanical damage before treatment [7].

This study has some limitations that should be considered. First, as a technical description, it primarily presents the surgical steps and rationale of the procedure rather than providing comparative clinical evidence. Accordingly, it does not establish superiority over isolated in situ fixation or open hip-preserving procedures. Another limitation is the high technical demand of hip arthroscopy in this setting, particularly given the need for precise portal placement, fluoroscopic control, and careful preservation of the retinacular vessels. Finally, although the postoperative protocol and imaging follow-up are described, longer-term clinical, radiographic, and functional outcomes are still necessary to determine the reproducibility, safety, and durability of this approach. Taken together, these considerations suggest that arthroscopic osteoplasty associated with in situ fixation may represent a potential technical option in carefully selected cases in which the goal is to control impingement while preserving physeal stability and vascular integrity. Future studies with larger cohorts and longer follow-up are needed to evaluate its safety, functional outcomes, and ability to prevent early degenerative changes.

## Conclusions

Arthroscopic hip-preserving treatment associated with in situ fixation represents a potential surgical approach for acute Grade I SCFE, allowing correction of cam morphology while preserving physeal stability and the vascular structures of the femoral head. By combining definitive stabilization of the slip with immediate treatment of the residual metaphyseal prominence, this technique addresses both the physeal and mechanical components of the deformity during the same procedure. In carefully selected low-grade cases, this approach is intended to address residual femoroacetabular impingement while avoiding the greater surgical morbidity associated with open reconstructive procedures. The key message of this technique is that even mild SCFE may benefit from early restoration of the head-neck contour when performed under direct arthroscopic and fluoroscopic control with strict attention to vascular preservation.
